# The Role of Active Site Hydrophobic Interactions in Facilitating Catalysis in Human Terminal Deoxynucleotidyl Transferase

**DOI:** 10.3390/ijms27010178

**Published:** 2025-12-23

**Authors:** Svetlana I. Senchurova, Timofey E. Tyugashev, Nikita A. Kuznetsov

**Affiliations:** 1 Institute of Chemical Biology and Fundamental Medicine, Siberian Branch of Russian Academy of Sciences, 630090 Novosibirsk, Russia; senchurova@1bio.ru (S.I.S.); tyugashev@1bio.ru (T.E.T.); 2 Department of Natural Sciences, Novosibirsk State University, 630090 Novosibirsk, Russia

**Keywords:** terminal deoxynucleotidyl transferase, protein–DNA interaction, nucleotide incorporation, molecular dynamics, template-independent DNA synthesis

## Abstract

Terminal deoxynucleotidyl transferase (TdT) is a unique DNA polymerase that catalyzes template-independent nucleotide addition at the 3′-end of DNA, playing a critical role in generating immune receptor diversity. While the structural importance of Loop1 in blocking template strand binding and enabling this activity is established, the precise molecular contribution of hydrophobic interactions within Loop1 to the catalytic mechanism of human TdT remains unclear. In the present study, we aim to elucidate the roles of hydrophobic Loop1 residues (L397, F400, F404) in the structural organization and catalytic function of TdT. We engineered alanine and tryptophan substitutions at these positions and systematically analyzed the resulting mutant forms using molecular dynamics simulations and pre-steady-state kinetic measurements. Our results show that substitutions L397A and F400A increase Loop1 flexibility and significantly reduce catalytic activity, particularly for purine nucleotide incorporation, while F404A completely abolishes enzymatic function. The F404W mutant largely preserves activity. All mutant forms retain the ability to bind single-stranded DNA and dNTP, but in some cases, their affinity and thermal stability were reduced. These findings demonstrate that hydrophobic interactions in Loop1 are essential for maintaining the catalytically competent conformation of TdT, ensuring precise substrate positioning and active site stability.

## 1. Introduction

DNA polymerases are enzymes responsible for DNA synthesis and repair, providing the faithful transmission of genetic information and the maintenance of genome integrity. They are essential for processes such as replication, recombination, and DNA repair, thereby supporting cell viability and preventing mutations and genetic disorders. Although all DNA polymerases catalyze nucleotide chain synthesis, they differ in their biological roles, substrate specificity, fidelity, and processivity, reflecting the diversity in their structures and mechanisms of action [1].

Based on amino acid sequence homology and structural features, DNA polymerases are classified into six major families: A, B, C, D, X, and Y [2,3]. In mammals, the X family comprises four enzymes: Pol β, Pol μ, Pol λ, and terminal deoxynucleotidyl transferase (TdT) [4]. These enzymes mainly function in various DNA repair pathways. Pol β is a central participant in base excision repair (BER) [5] a cellular repair pathway, which is responsible for recognition and excision non-bulky damaged bases from DNA while Pol μ and Pol λ are involved in repairing double-strand breaks through non-homologous end joining (NHEJ) a crucial but error-prone repair pathway that directly ligates broken DNA end without of homologous template [6]. Pol λ also serves as a backup polymerase in the BER pathway [7]. Notably, Pol β and Pol λ possess dRP lyase activity, which facilitates a β-elimination reaction to cleave the DNA backbone and excise the 5′-deoxyribose phosphate (5′-dRP) fragment generated by AP endonuclease during BER [8].

In contrast to these template-dependent repair polymerases, TdT occupies a special position within the X family due to its unique template-independent activity. Unlike conventional DNA polymerases, TdT randomly adds nucleotides to the 3′-hydroxyl end of single-stranded DNA without a template. The primary biological function of TdT is to generate diversity in antigen receptor genes during V(D)J recombination in lymphocytes. During the joining of gene segments, TdT adds from 1 to 10 random nucleotides (N-nucleotides) to 3′-ends, increasing the variability of antigen-binding regions in immunoglobulins and T-cell receptors, which is fundamental for the adaptive immune system [9,10,11,12,13].

Structurally, TdT shares the thumb, fingers, and palm subdomains found in all DNA polymerases and contains two divalent metal ions in the active site [14]. It operates via a two-metal ion mechanism: metal A activates the 3′-end of the DNA primer for nucleophilic attack, while metal B stabilizes the pyrophosphate leaving group [15]. These ions are coordinated by oxygen atoms from the dNTP and the carboxyl groups of three amino acid residues—D343, D345, and D433 [14]. TdT can utilize a range of divalent metal ions, including Mg^2+^, Mn^2+^, Co^2+^, and Zn^2+^. In vitro studies have shown that the nature of the metal cofactor affects the rate of nucleotide incorporation by TdT [16,17].

A distinctive structural feature of TdT is Loop1, located in the palm subdomain, which sterically blocks the binding of duplex DNA in the active site, occupying the space where the template strand would be positioned in template-dependent polymerases [14]. The dNTP-binding pocket, partially formed by Loop1, is composed of hydrophobic residues (L397, F404, W449) on one side and hydrophilic residues (R336, H342, D395, R453, E456, R457) on the other [14,18]. Molecular dynamics simulations have shown that the hydrophilic residues form a network of hydrogen bonds with the bases of incoming nucleotides [17]. Mutational analysis of D395 and E456 has demonstrated that altering, repositioning, or neutralizing their side chain charges affects substrate specificity of TdT for natural nucleotides; the double substitution D395N/E456N notably increases the incorporation rate for all natural nucleotides [19,20].

The functional roles of murine TdT L398A, F401A, and F405A mutant forms, corresponding to human TdT L397A, F400A, and F404A, have been examined in several studies. In crystal structures of the wild-type (WT) murine TdT, the L398 side chain is inserted between the 3′-terminal and 3′-penultimate bases of the DNA primer, oriented toward the F405 side chain. This hydrophobic wedge disrupts the π-π stacking interaction in the DNA primer and forces the 3′-terminal base to stack with the incoming dNTP. The L398A mutation was initially found to abolish Mg^2+^-dependent terminal transferase activity, with Co^2+^ partially restoring it, although subsequent work showed a reduced, but notable level of non-templated activity with Mg^2+^ [18,21]. F401 forms hydrophobic contacts with F384, maintaining the correct position of Loop 1 [18]. The F401A variant was originally noted to display only template-dependent polymerase activity, with transition metals unable to restore terminal transferase activity, but later studies observed both template-dependent and template-independent activity in the presence of Mg^2+^. The F405A variant showed limited template-dependent polymerase activity in Mg^2+^, with terminal transferase activity restored by CoCl_2_. Crystal structures of F401A and F405A mutant forms bound to DNA double-strand mimics revealed increased disorder in the Loop1 region and at the primer 3′ terminus compared to the WT protein [21,22].

Recently, mTdT F405Y and F405H mutant forms have been examined for their affinity for ssDNA and ssRNA primers [23]. F405Y was observed to display both reduced activity on ssDNA primers, processing less than 20% of the ssDNA primer and extending them only for up to ~50 nt, compared to more than 1000 nt for WT TdT under the same conditions, while on ssRNA substrate, its reaction yield was improved to 57% with extension lengths up to 100 nt compared to 44% yield with up to 50 nt addition products of the WT enzyme. F405H displayed no ssDNA activity and reduced ssRNA activity.

Given the critical role of hydrophobic Loop1 residues in the unique template-independent activity of TdT and the evidence that their substitution alters substrate specificity and processivity, we focused in this study on the L397A, F400A, F404A, and F404W mutant forms. By integrating molecular dynamics simulations with pre-steady-state kinetic analysis, we aimed to link specific structural and dynamic perturbations in the Loop1 region to changes in the catalytic function. This combined approach provides a mechanistic understanding of how hydrophobic residues L397, F400, and F404 dictate the template-independent activity and substrate specificity of TdT.

## 2. Results and Discussion

### 2.1. MD Simulations

**Figure 1:**
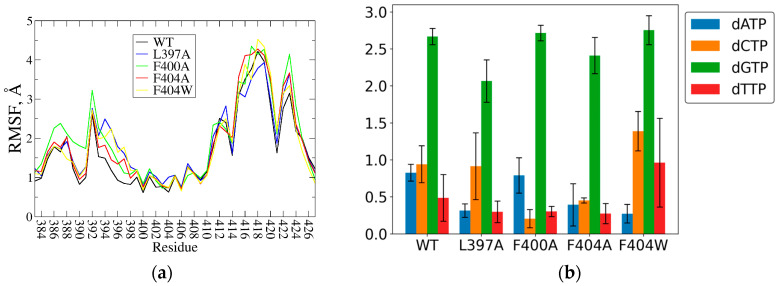
Global effect of the amino acid substitutions in simulations of the hTdT pre-catalytic complex: (**a**) RMSF of residues in Loop1 (384–400) and Loop2 (412–426) regions in simulations of TdT-ssDNA-dGTP complexes. (**b**) Average number of hydrogen bonds between the base of the incoming dNTP and the protein amino acid residues, error bars represent the standard error of the mean for independent replicas.

In this study, we employed molecular dynamics simulations to perform a functional analysis of three hydrophobic amino acid residues—L397, F400, and F404—in both pre-catalytic and post-catalytic protein–DNA complex states. Simulations of the TdT-ssDNA-dNTP complex structures show an overall increase in flexibility of the protein structure motile elements, including both Loop1 and Loop2 (Figure 1a). Unlike our previous mutational studies of active site residues directly engaging the nucleobase of the incoming dNTP, and with the exception of the L397A variant, increased flexibility of the primer- and dNTP-facing Loop1 region is mostly unreflected in hydrogen bonding between the protein and the incoming base (Figure 1b).

#### 2.1.1. Role of L397

**Figure 2:**
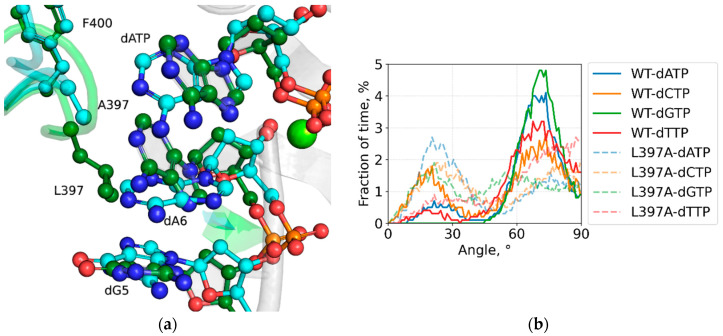
Simulation results for the L397A pre-catalytic complex: (**a**) Overlay of simulation snapshots for WT-ssDNA-dATP and L397A-ssDNA-dATP complexes. Selected residues and Loop1 backbone are highlighted in green for WT and in cyan for L397A, and light green indicates the Mg^2+^. (**b**) Distribution of angle between planes of 3′-terminal and 3′-penultimate residues.

In molecular dynamics simulations of the TdT WT, the 3′-terminal (dA6) and 3′-penultimate (dG5) bases are predominantly split apart by the wedging action of the L397 sidechain, though alternate states with the L397 sidechain everted out are also present. Simulations of the L397A mutant form complexes show that the shortened aliphatic side chain diminishes this wedging effect, generally allowing the bases of the DNA primer terminus to reestablish π-π stacking interactions, indicated by reduced interplanar angle (Figure 2a,b). And if larger purine bases of the incoming dNTP follow the primer 3′-terminal base via their base stacking contact, they lose previously established hydrogen bonds at the protein active site, mostly evident in loss of hydrogen bonds between the incoming guanine base and sidechains of theR453, E456, R457 active site residues (Figure 1b). Competent binary and post-catalytic binary complex models display similar effects.

Loss of the wedging interaction with the DNA strand is also reflected in increased motility of the dNTP-interacting segment of Loop1, while not affecting the more distant Loop2 region (Figure 1a).

#### 2.1.2. Role of F400 and F404

**Figure 3:**
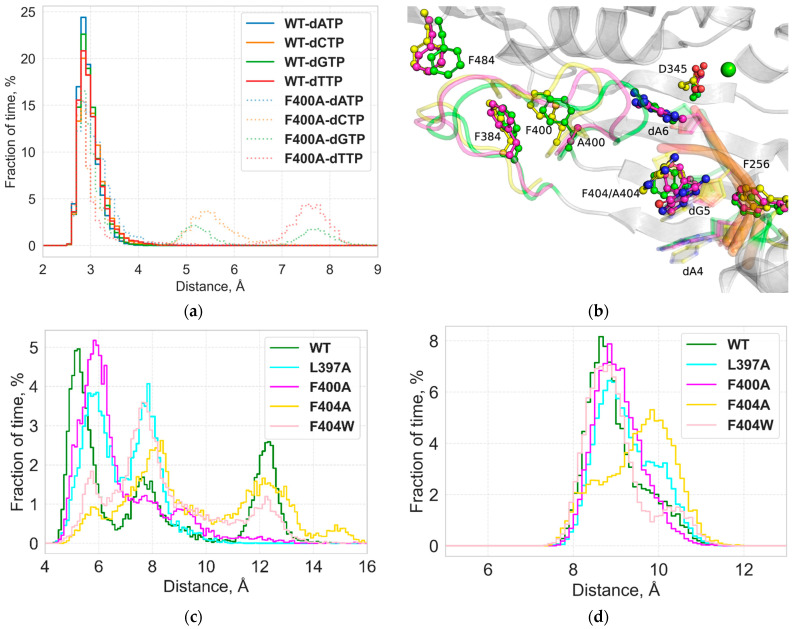
Simulation results of the F400A and F404A amino acid substitutions: (**a**) Distribution of distances between the Nδ atom of the H474 residue and the Oδ1 atom of the D472 residue in the SD2 region in the pre-catalytic complex. (**b**) Overlay of simulation snapshots for WT-ssDNA, F400A-ssDNA, and F4004A-ssDNA complexes. Selected residues and Loop1 backbone are highlighted in green for WT, in yellow for F404A, and in magenta for F400A, and light green indicates the Mg^2+^. (**c**) Distribution of distances between the 3′OH group of the 3′-terminal dA6 base and the Cα atom of the D345 active site residue in the competent binary complex. (**d**) Distribution of distances between Cβ atoms of the F256 (fingers domain) and F404/A404 (palm domain) residues in the competent binary complex.

Simulations of the ternary F400A and F404A/W TdT-ssDNA-dNTP complexes maintain the same active site structure while displaying an overall increase in RMSF of motile elements, including both Loop1 and Loop2 regions (Figure 1a), as well as the 5′ terminus of the DNA primer. The F400A substitution loosens the hydrophobic core of the Loop1 region originally anchored by a π-π stacking interaction between the F400 and F384 sidechains, resulting in an increase in flexibility of the solution-facing Loop1 region 384–392 (Figure 1a). The void created by the shorter A400 sidechain allows the H474 sidechain to reposition, breaking a previously stable hydrogen bond between the D472 and H472 residues (Figure 3a), both belonging to a highly conserved TdT SD2 motif [22]. Both substitutions of the F404 sidechain provoke minor destabilization of their environments in the ternary complex models. However, the effects of these substitutions are far more pronounced in models of the binary competent state, where the active site lacking both dNTP and the Metal A ion allows for more flexible positioning of the DNA 3′ terminus and Loop1 (Figure 3b). Both F400A and F404A complex modes show increased displacement of the DNA primer and Loop1 away from the active site compared to the WT complex (Figure 3c).

The F404A substitution turns out to be more destabilizing for the protein-DNA complex, displaying both the lowest number of hydrogen bonds and the highest MMPBSA estimate of relative binding free energy across the simulation time, as well as an increase in distance between the fingers and palm domains in the otherwise stable fold of the polymerase (Figure 3d, Table 1).

#### 2.1.3. MD Simulation of F404W

**Figure 4:**
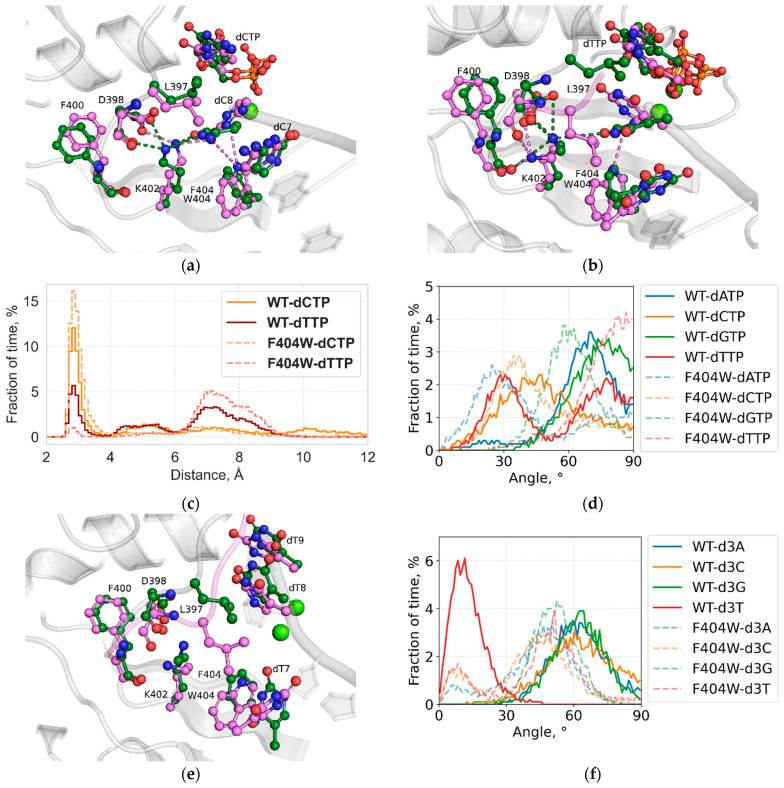
Simulation of the extended pre-catalytic and post-catalytic complexes of the F404W variant: (**a**) Overlay of simulation snapshots for WT-ssDNA + 2dC-dCTP and F404W-ssDNA + 2dC-dCTP complexes. Selected residues and Loop1 backbone are highlighted in green for WT and in pink for F404W, salt bridges between amino acid sidechains and the sugar–phosphate backbone are shown as dashed lines, and light green indicates the Mg^2+^. (**b**) Overlay of simulation snapshots for WT-ssDNA + 2dT-dTTP and F404W-ssDNA + 2dT-dTTP complexes. Selected residues and Loop1 backbone are highlighted in green for WT and in pink for F404W, salt bridges between amino acid sidechains and the sugar–phosphate backbone are shown as dashed lines and light green indicates the Mg^2+^. (**c**) Distribution of distance between the K402 NZ atom and the dCTP/dTTP O2 atom for WT and F404W dCTP and dTTP in the pre-catalytic elongated primer complexes. (**d**) Distribution of angle between planes of 3′-terminal and 3′-penultimate residues in the pre-catalytic elongated primer complexes. (**e**) Overlay of simulation snapshots for WT-ssDNA + 3dT and F404W-ssDNA + 3dT elongated product complexes. Selected residues are highlighted in green for WT and in cyan for F404W, and light green indicates the Mg^2+^. (**f**) Distribution of angle between planes of the F404/W404 sidechain and the antepenultimate base (dN7) in the post-catalytic elongated primer complexes.

Substitution of the F404 sidechain by tryptophan introduces a new hydrogen donor group into the position that previously served as a fully hydrophobic “strike plate” for the L397 “pin” of the L397-F404 latch. Relative positioning of the W404 sidechain results in the formation of a new persistent H-bond between its Nε1 atom and the O_4_′ atom of the 3′-terminal primer nucleotide. Surprisingly, while for both thymine and cytosine, the O_2_ atom could be expected to act as another acceptor of this hydrogen, during the molecular dynamics simulation, this occurrence is observed only for the cytosine-capped primer, with the 3′ cytosine base turning away from the cytosine base of the incoming dCTP (Figure 4a,b).

In another contrast between the two pyrimidine bases, the substitution also differentially affects the hydrogen bond network centered on the K402 residue. In the WT protein complexes, both in available crystal structures and in molecular dynamics simulations with 3′-terminal purine bases, K402 forms hydrogen bonds with the backbone carbonyl oxygens of D398 and F400, as well as with the sidechain carboxylate of D398. However, in MD simulations of TdT WT bound to a pyrimidine-capped primer, K402 additionally engages the O_2_ atom of both cytosine and thymine. This interaction displaces the sidechain of L397 from its wedge position and reorients the 3′-terminal primer base away from the incoming dNTP base. For the WT protein, this effect provides another plausible explanation for its relatively slower transferase activity with pyrimidine dNTPs. Simulations of the F404W substitution complexes show that for the F404W-ssDNA + 2dC-dCTP elongated primer ternary complex this alternate bonding network of K402 becomes even more pronounced, reflected both in decreased distance between the K402 NZ and O_2_ atoms indicating increased share of states with H-bonds present between the sidechain’s amino group and hydrogen acceptor atoms of the 3′-terminal dC base (Figure 4c), as well as shallower angle between the terminal and the penultimate bases (Figure 4d), representing decrease in the L397 wedge effect. Quite the opposite, in the F404W-ssDNA + 2dT-dTTP complex, the number of states with the hydrogen bond between the K402 sidechain and the terminal dT base is sharply reduced, and the L397 is more present in the wedged state.

Another possible impact of the F404W substitution for dT incorporation emerges in the elongated primer post-catalytic state simulations. In the WT extended product complex, bases at the antepenultimate primer position, proximal to the F404 sidechain, lack stacking interactions in both crystal structures and simulations, except for the dT complex, where the dT7 base (Figure 4e) maintains a π-π stacking interaction with the F404 sidechain. In the F404W extended product complex, this interaction is disrupted by the tryptophan substitution. The dT base adopts an interplanar angle similar to that of the other bases, likely due to the larger indole ring of tryptophan, resulting in a steric clash disrupting the original stacking contact. A slight reduction in interplanar angle is also observed for the other three bases in the F404W complexes relative to their WT counterparts (Figure 4f).

Thus, the modeling predicted that amino acid substitutions disrupt local and global active site stability in different ways. To test these predictions, we proceeded to experimental characterization of the protein variants.

### 2.2. Structural and Binding Characterization of TdT Mutant Forms

To bridge the gap between computational prediction and biological function, we proceeded to test the key hypotheses generated by our MD simulations. We constructed the corresponding mutant forms (L397A, F400A, F404A, F404W) and performed a focused experimental characterization of their activity. The simulations suggested varying degrees of structural perturbation. To assess this at a global level, we examined the secondary structure and thermal stability of each variant. Concurrently, since a catalytically competent state requires precise substrate binding, we quantitatively evaluated the affinity of each mutant form for both a single-stranded DNA primer and a nucleotide substrate. This combined analysis allowed us to determine whether the substitution primarily affects the enzyme’s scaffold or its direct interaction with substrates.

#### 2.2.1. Impact of the Introduced Amino Acid Substitutions on the Protein Secondary Structure

**Figure 5:**
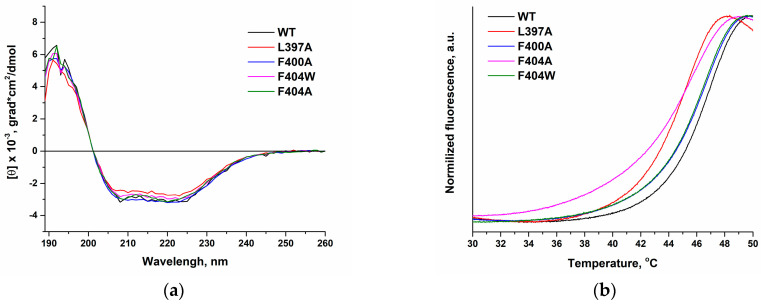
Experimental characterization of the secondary structure of TdT mutant forms compared to the WT enzyme: (**a**) Circular dichroism spectra of TdT WT and the L397A, F400A, F404A, and F404W mutant forms. [TdT] = 2 µM, 25 °C. (**b**) Melting curves of TdT WT and the L397A, F400A, F404A, and F404W mutant forms. [TdT] = 10 µM.

The impact of introduced amino acid substitutions on the secondary structure of the protein was assessed using circular dichroism (CD) spectroscopy and thermal denaturation measurements (T_m_) (Figure 5). The CD spectra obtained for all mutant variants were essentially indistinguishable from that of the WT enzyme, indicating that these substitutions do not perturb the protein’s secondary structure. The thermal stability of the protein globule, as determined by thermal denaturation measurements, reflects the relative stability of the mutant forms compared to the WT enzyme. The data obtained indicate that the melting temperatures (T_m_) for variants F400A and F404W matched that of the WT enzyme, whereas substitutions L397A and F404A resulted in a 1.5–2 °C decrease in T_m_ (Table 2). Thus, the local alterations introduced by F400A and F404W have a negligible effect on the overall secondary structure, while the L397A and F404A substitutions cause a modest destabilization of the protein fold. Overall, the secondary structure remains largely intact. However, the increased molecular flexibility associated with the latter substitutions makes the protein more susceptible to thermal denaturation.

#### 2.2.2. Impact of the Introduced Modifications on DNA and Flu-dUTP Binding Efficiency

**Figure 6:**
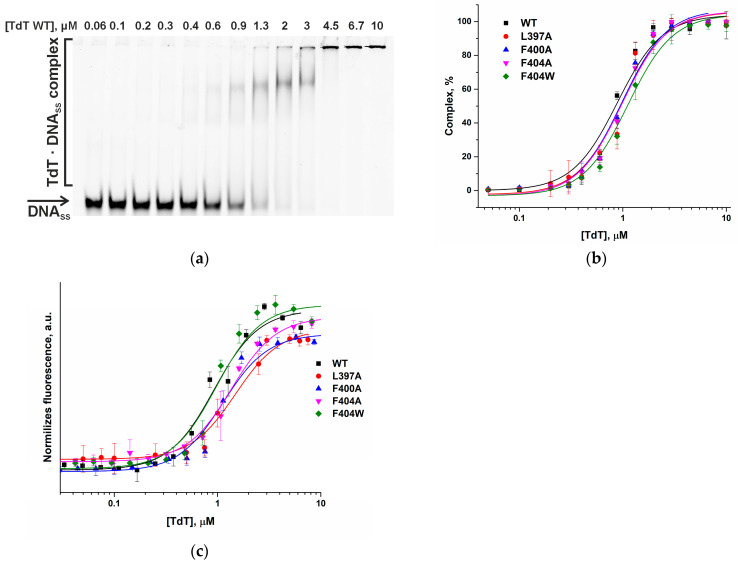
Binding analysis of DNA primer FAM-M44 and dNTP (Flu-dUTP) with TdT mutant forms: (**a**) Example of EMSA showing binding of mutant TdT WT to FAM-M44 in the presence of Mg^2+^. (**b**) Titration curves of FAM-M44 binding for TdT mutant forms, enzyme concentrations ranging from 0.06 to 10 µM, with 0.5 µM FAM-M44. (**c**) MST titration curves illustrating interactions between WT and mutant TdT forms with Flu-dUTP in Mn^2+^ presence. Enzyme concentrations ranging from 0.05 to 15 µM, with 0.5 µM Flu-dUTP. Experimental data are shown as points: black (■) for WT, red (•) for L397A, blue (▲) for F400A, magenta (▼) for F404A, and green (◆) for F404W. The corresponding colored curves represent the theoretical fit.

The effect of specific amino acid substitutions on the affinity of TdT for the single-stranded DNA primer and incoming dNTP was evaluated by determining the corresponding dissociation constants (*K*_d_). The dissociation constant (*K*_d_) quantifies binding affinity, where a lower value indicates tighter binding. Electrophoretic mobility shift assay (EMSA) was employed to characterize the binding of TdT to a 44-nucleotide single-stranded DNA primer FAM-M44 (Figure 6a,b). The dissociation constants were derived using Equation (2) (Table 3). The determined *K*_d_ for the mutant and WT enzymes revealed that all analyzed substitutions had only a minor effect on DNA primer binding. Affinity decreased slightly in the following order: WT ≈ L397A ≈ F400A ≈ F404W > F404A (Figure 6a,b, Table 3). This result indicates that none of the studied substitutions disrupts critical protein contacts with the DNA primer.

The affinity of the TdT variants for the fluorescent nucleotide analog (Flu-dUTP) was measured by microscale thermophoresis (MST) (Figure 6c). TdT efficiently incorporates this nucleotide, despite the fact that it is modified with a large hydrophobic fluorescent group [24]. The data revealed no significant impairment of substrate binding, with the affinity decreasing in the series: WT ≈ F404W ≈ F400A > L397A ≈ F404A (Table 3). Aromatic side chains at F404 and F400 contribute to hydrophobic and π-π stacking interactions within the dNTP-binding pocket. The F404A substitution removes this aromatic interaction, explaining the decrease in affinity, while F404W maintains or even enhances aromatic stacking, preserving affinity. The negligible effect of F400A on *K*_d_ supports the MD observation that F400 does not form critical direct contacts with the dNTP, or that its role can be compensated by neighboring residues. The L397A substitution, while primarily affecting DNA primer positioning, also modestly reduces dNTP affinity. This is consistent with MD simulations showing that L397A increases Loop1 flexibility, which may slightly destabilize the overall architecture of the nucleotide-binding pocket without abolishing direct contacts.

These experiments confirmed the structural changes predicted by MD simulations, with the F404A substitution proving the most pronounced effect. Importantly, all mutant forms retained the ability to bind both DNA and Flu-dUTP. Since binding is maintained, loss of function likely occurs later: during precise positioning of molecules for the chemical reaction or during the catalytic reaction itself.

### 2.3. dNTP Incorporation Efficiency

Given that MD simulations indicated defects in active site organization while binding studies confirmed stable substrate interactions, we next directly assessed the catalytic efficiency of the mutant enzymes.

**Figure 7:**
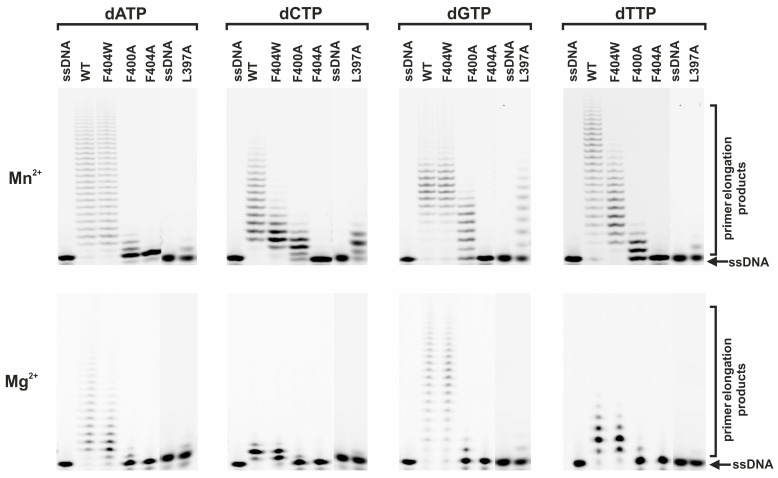
Comparison of the dNTP incorporation efficiency by WT TdT and the L397A, F400A, F404A, and F404W mutant forms in the presence of Mg^2+^ or Mn^2+^ ions. Reaction conditions: [TdT] = [DNA] = 1.0 µM, [dNTP] = 10 µM, reaction time = 5 min.

To evaluate the impact of amino acid substitutions on dNTP incorporation efficiency, we measured product accumulation catalyzed by mutant forms of the enzyme in the presence of Mg^2+^ or Mn^2+^ cofactors (Figure 7). In the presence of the native Mg^2+^ ion, all mutant forms exhibited reduced catalytic activity. The F404W variant retained the highest residual activity, consistent with the preservation of hydrophobicity and aromatic character in the dNTP-binding pocket that apparently is critical for substrate binding. The slight activity decrease may stem from steric hindrance introduced by the bulkier tryptophan side chain, which induces local alterations in active site architecture. In stark contrast, the F404A substitution abolished all enzymatic activity with both Mg^2+^ and Mn^2+^. This complete loss of function underscores the essential role of an aromatic residue at position 404 in maintaining active site integrity, where the elimination of hydrophobic and stacking interactions fully destabilizes the substrate-binding pocket. The F400A and L397A mutant forms also showed significantly diminished activity with Mg^2+^. For F400A, the reduced activity likely results from increased flexibility in Loop 1, which compromises the optimal organization of the active site without completely preventing substrate binding. The severe impairment of the L397A mutant, conversely, points to a critical role for this residue in ensuring the precise positioning of the primer’s 3′ terminus, a prerequisite for efficient catalysis.

Notably, Mn^2+^ ions partially restored activity for the F404W, F400A, and L397A mutant forms but had no effect on the F404A variant. This partial rescue is consistent with the known tolerance of Mn^2+^ for deviations in active site geometry, allowing it to compensate for moderate structural perturbations. Inability of F404A to restore activity underscores the absolute requirement for a stable binding pocket, the structure of which is disrupted by this mutation. In summary, the catalytic profiles of the mutant forms correspond to the defined structural roles of these residues, confirming their importance in active site stabilization and substrate binding.

#### 2.3.1. dNTP Incorporation by the L397A, F400A, and F404W Mutant Forms in the Presence of Mn^2+^

**Figure 8:**
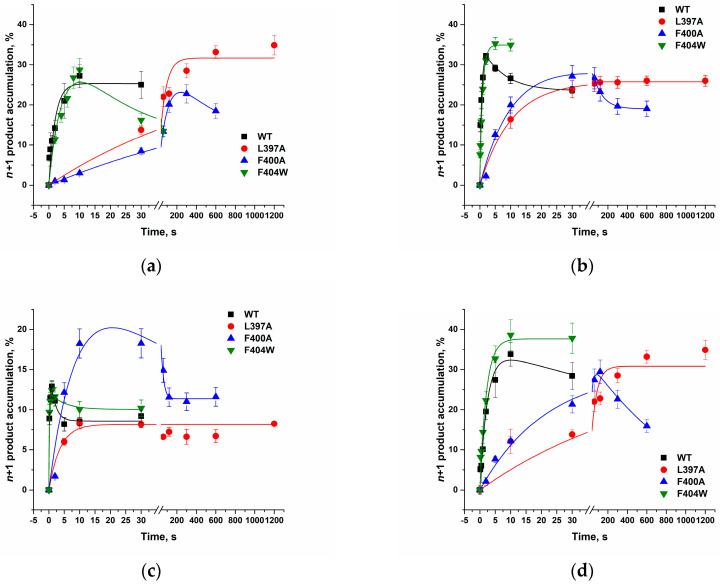
Kinetics of dNTP incorporation by TdT and its mutant forms in the presence of Mn^2+^. Time courses for the incorporation of dATP (**a**), dCTP (**b**), dGTP (**c**), or dTTP (**d**) by TdT WT, L397A, F400A, and F404W. Reaction conditions: 50 mM Tris–HCl (pH 8.0), [TdT] = [primer] = 1.0 μM, [dNTP] = 2.0 μM, [Mn^2+^] = 1.0 mM. Experimental data are shown as points: black (■) for WT, red (•) for L397A, blue (▲) for F400A and green (▼) for F404W. The corresponding colored curves represent the theoretical fit.

**Figure 9:**
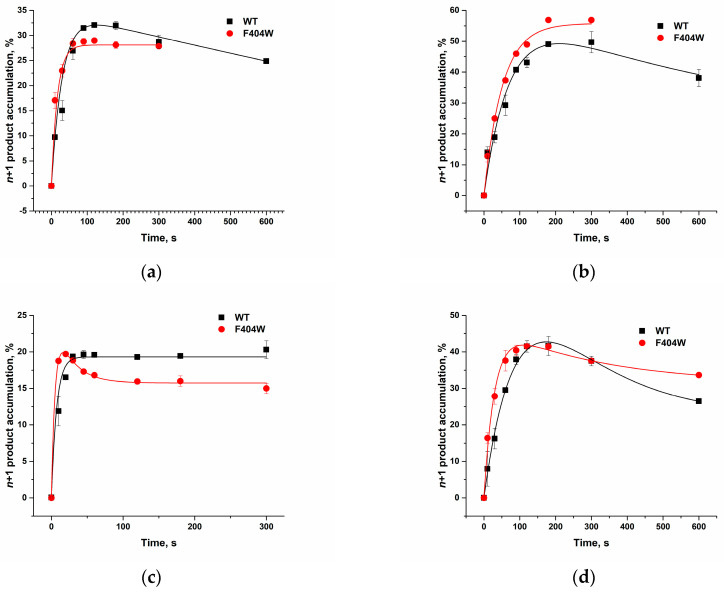
Kinetics of dNTP incorporation by TdT WT and F404W in the presence of Mg^2+^ ions. Time courses for the incorporation of dATP (**a**), dCTP (**b**), dGTP (**c**), or dTTP (**d**) by TdT WT and F404W. Reaction conditions: 50 mM Tris–HCl (pH 8.0), [TdT] = [primer] = 1.0 μM, [dNTP] = 2.0 μM, [Mg^2+^] = 5.0 mM. Experimental data are shown as points: black (■) for WT, red (•) for F404W. The corresponding colored curves represent the theoretical fit.

To assess the impact of amino acid substitutions on the entire catalytic cycle, including both the stages of enzyme binding to the DNA primer and the binding and incorporation of the inserted nucleotide, an analysis of the kinetics of *n* + 1 product accumulation was carried out (Figure 8 and Figure 9). In the presence of Mn^2+^ ions, substitution of the analyzed amino acid residues with alanine resulted in reduced incorporation rates for all dNTPs compared to the WT enzyme, whereas the F404W substitution caused only a minor decrease in the incorporation rate specifically for dCTP (Table 4). The nucleotidyl transferase reaction rate for natural substrates in WT enzyme decreased in the order: dGTP > dCTP >> dATP ≈ dTTP. All mutant forms largely retained specificity toward natural dNTPs, with the highest incorporation rates observed for dCTP and dGTP.

The most pronounced changes in specificity were observed for the L397A mutant. In this variant, the dGTP incorporation rate decreased so substantially that it became comparable to the incorporation rates of dATP and dCTP, resulting in the greatest overall reduction in catalytic efficiency (10- to 40-fold for different nucleotides). This suggests that the L397 residue is critical for maintaining the rigidity of the dNTP-binding pocket, and its substitution disrupts the precise positioning of the incoming nucleotide’s nitrogenous base, an effect particularly pronounced for purines. A similar, though less pronounced, effect was observed for the F400A substitution, which also most strongly affected the incorporation of purine nucleotides (dATP and dGTP). This indicates an important role for the F400 residue in orienting and stabilizing the substrate’s nitrogenous base within the active site. In stark contrast, the F404A mutation completely abolishes catalytic activity, underscoring the indispensable structural role of this aromatic residue for active site integrity. Conversely, the F404W mutant largely preserves activity but exhibits an altered substrate specificity profile. This functional shift aligns with MD simulations, indicating that the tryptophan substitution modifies local interaction networks, which can differentially affect the processing of various nucleotides. Thus, residues L397, F400, and F404 are critical for the overall efficiency of incorporation of all nucleotides.

#### 2.3.2. dNTP Incorporation by the F404W in the Presence of Mg^2+^

We also evaluated the catalytic activity of the F404W mutant form in the presence of Mg^2+^ ions (Figure 9). Mg^2+^ ions require a strict geometry of the active site, in contrast to the more flexible coordination sphere tolerated by Mn^2+^. Therefore, using Mg^2+^ as a cofactor can reveal the precise structural consequences of amino acid substitutions on catalysis, which may be masked in the presence of the more promiscuous Mn^2+^. A comparison of the observed rate constants demonstrated that the tryptophan substitution increases the rate of thymidine dTTP incorporation approximately threefold and the rate of adenosine dATP incorporation by 1.5-fold (Table 5). Our molecular dynamics simulations showed that the F404W substitution creates a new hydrogen bond and alters the interaction network with K402, differentially affecting the conformation of the primer with dC and dT at the 3′ end. The observed threefold increase in the rate for dTTP is consistent with the new active site conformation in the F404W mutant being particularly favorable for the positioning and/or chemical step of thymidine incorporation. The more moderate effect for dATP (1.5-fold) suggests that the purine recognition or stacking mechanism is less sensitive to this change. Thus, it can be hypothesized that F404 acts as a “switch” for TdT substrate specificity. The F404W mutation fine-tunes the active site, shifting its preference toward pyrimidines, particularly dTTP, under stringent conditions (Mg^2+^). This provides a clear mechanistic rationale for the change in catalytic profile observed in Figure 9 and Table 5.

## 3. Materials and Methods

### 3.1. Enzyme and Purification

Mutations in the TdT gene were introduced by PCR-based site-directed mutagenesis using Pfu DNA polymerase and specific primer pairs (Table 6), following a standard protocol [19]. The resulting product was treated with Mal I (Sibenzyme, Novosibirsk, Russia) to remove the parental template and transformed into *E. coli* XL1Blue cells. All constructs were verified by DNA sequencing.

The recombinant proteins were expressed and purified following a previously published method [17] using a two-step chromatography protocol. Briefly, clarified lysate from cells disrupted by French press was applied to a Ni affinity column HiTrap™ Chelating HP (Cytiva, Washington, DC, USA), and the target protein was eluted with a linear imidazole gradient 30 → 500 mM in 20 mM HEPES-KOH pH 7.8 m, 500 mM NaCl. The pooled fractions were diluted in 20 mM HEPES-KOH, with pH 7.8, to NaCl at a final concentration of 200 mM and further purified by ion exchange chromatography using a HiTrap™ Heparin HP column (Cytiva, Washington, DC, USA) with elution via a NaCl gradient 200 → 1000 mM in 20 mM HEPES pH 7.8. As a final step, the protein was dialyzed into storage buffer comprising 20 mM HEPES, 1 mM DTT, and 200 mM NaCl, pH 7.8, supplemented with glycerol to a final concentration 50% (*v*/*v*), and stored at −20 °C.

### 3.2. Oligonucleotides

The oligonucleotides used in this study were purchased from Biosset (Novosibirsk, Russia). Oligonucleotides were synthesized on an ASM-800 automated DNA/RNA synthesizer (Biosset, Novosibirsk, Russia) using commercial 2′-deoxyribonucleoside phosphoramidites and CPG carriers (GlenResearch, Sterling, VA, USA) (Table 7).

### 3.3. Circular Dichroism (CD) Spectroscopy

CD spectra were recorded using a Jasco J-600 spectropolarimeter (Jasco, Tokyo, Japan) at a wavelength of 180 to 260 nm in a quartz cuvette with a 1 mm optical path length. The protein concentration in the cuvette was 2 µM. Experiments were conducted in 50 mM Tris-HCl buffer (pH 8.0) containing 5 mM MgCl_2_ at 25 °C. Spectra were acquired with a bandwidth of 1.0 nm. Scans were accumulated and automatically averaged.

### 3.4. Melting Temperature (Tm) Measurements

Melting temperature (Tm) was determined using a QuantStudio™ 5 Real-Time PCR system (Applied Biosystems, Waltham, MA, USA). Each reaction tube contained protein (10 µM), 50 mM Tris-HCl (pH 8.0), 5 mM MgCl_2_, and 5X ProteOrange dye (Lumiprobe, Moscow, Russia). The temperature was gradually increased from 25.0 °C to 99.9 °C at a rate of 0.015 °C/s. The excitation and emission wavelengths for ProteOrange were 470 nm and 588 nm, respectively. Three replicate fluorescence intensity curves were recorded for each protein, then averaged and normalized. Tm values were calculated using OriginPro 15.0 software (OriginLab, Northampton, MA, USA) by fitting the data to the Boltzmann sigmoidal equation:

*F* = F_u_ + (F_b_ − F_u_)/[1 + exp(T_m_ − *T*/slope)], (1)

where *F*—ProteOrange fluorescence intensity, F_u_—initial fluorescence intensity (at 25 °C), F_b—_maximum fluorescence intensity (at 99.9 °C), T_m_—melting temperature, *T*—temperature, and slope—curve steepness.

### 3.5. Determination of DNA-Primer Binding Parameters

The binding affinity of TdT and its mutant variants for the single-stranded DNA primer (FAM-M44) was determined using gel shift assays. Proteins were serially diluted in reaction buffer (50 mM Tris-HCl, pH 8.0, 5 mM MgCl_2_, 7% glycerol). Equal volumes of 1 µM FAM-M44 in the same buffer were added. Final concentrations in the binding reactions for FAM-M44 were 0.5 µM, and for the proteins, they ranged from 0.06 µM to 10 µM. Samples were incubated for 10 min at 37 °C, and then 5 min at 0 °C for complex stabilization. Gel electrophoresis was performed for 40 min at 150 V and 4 °C. The resulting gels were visualized in the VersaDoc gel-documenting system (Bio-Rad Laboratories, Inc., Hercules, CA, USA). The degree of protein complex formation with DNA was determined using the Gel-Pro Analyzer 3.1 software (Media Cybernetics, Rockville, MD, USA).

The dissociation constant *K*_d_ were calculated using the Hill equation using OriginPro 15.0 software (OriginLab, Northampton, MA, USA):

*Formed complex (%)* = F_u_ + (F_b_ − F_u_)/(1 + (*K*_d_/*[E]*_0_)h), (2)

where F_u_—background contribution, F_b_—intensity of the TdT-DNA complex band, *K*_d_—dissociation constant, *[E]*_0_—enzyme concentration, and h—Hill coefficient.

During data processing, the value of the Hill coefficient (h) was fixed at 2. This was necessary because the data could not be adequately fitted under the assumption of single-site cooperativity. This result can be explained by the hypothesis that the observed cooperativity arises from enzyme dimerization.

### 3.6. Determination of dNTP Binding Parameters

The effect of the introduced amino-acid substitutions on the binding of TdT to fluorescently labeled Flu-dUTP was determined using the Monolith NT.115 system (NanoTemper Technologies, Munich, Germany) using standard capillaries (Monolith^TM^ NT.115 capillaries with standard treatment). Each point on the titration curves was determined by measuring the fluorescence intensity of individual solutions (16 μL) containing Flu-dUTP (0.5 μM) and enzyme (0.05–15 μM) in buffer containing 50 mM Tris-HCl (pH 8.0) and 5 mM MnCl_2_ at 37 °C. The dissociation constant values, *K*_d_, were calculated using the Hill equation:

*F* = F_u_ + (F_b_ − F_u_)/(1 + (*K*_d_/*[E]*_0_)h), (3)

where *F*—Fluorescein fluorescence intensity, F_u_—Fluorescein fluorescence intensity in the absence of enzyme, F_b_—maximum fluorescein fluorescence intensity in the enzyme complex, *K*_d_—dissociation constant, *[E]*_0_—enzyme concentration, and h—Hill coefficient.

During data processing, the Hill coefficient (h) was fixed at 2. A potential explanation for the observed cooperativity in the interaction of TdT with Flu-dUTP is provided by its molecular model, which posits two binding centers. One center binds the 3′ end of the primer, while the other binds the dNTP. This is supported by the enzyme’s ability to perform de novo synthesis of polynucleotides (2–15 nucleotides in length) in the presence of dNTPs but the absence of a primer [25].

### 3.7. Nucleotidyl Transferase Activity Assay

The accumulation of natural nucleoside triphosphate addition products by TdT WT and its mutant forms was analyzed under pre-steady-state conditions. Reaction products were separated by denaturing polyacrylamide gel electrophoresis. Reactions were performed in 50 mM Tris-HCl (pH 8.0) containing 1 mM MnCl_2_ or 5 mM MgCl_2_ at 37 °C. To initiate the reaction, equal volumes of 2 μM enzyme and a solution containing 4 μM dNTP and 2 μM FAM-labeled DNA primer (FAM-M6) were mixed. Aliquots (10 μL) were taken at specified time points and quenched with an equal volume of stop solution (9 M urea, 25 mM EDTA, 0.1% xylene cyanol, 0.1% bromophenol blue). For time points shorter than 10 s, quenching was achieved using a KinTek RQF-3 Quench-Flow apparatus (KinTek Corp., Snow Shoe, PA, USA). The quenched samples were electrophoresed on 20% denaturing polyacrylamide gels at 300 V for 5 h. The extent of substrate conversion was quantified using Gel-Pro Analyzer 3.1 software (Media Cybernetics, Rockville, MD, USA) by calculating the ratio of the peak area for the *n* + 1 product to the sum of the peak areas for all products and the starting oligonucleotide. The observed rate constants for the formation and consumption of the *n* + 1 product were determined by fitting the data to the following equation:

*y* = A_1_ × [1 – exp(−*k*_1_ × *t*)] + A_2_ × [1 – exp(−*k*_2_ × *t*)], (4)

where A_i_—the amplitude (extent of product accumulation), k_i_—are the observed rate constants for accumulation and consumption of the *n* + 1 product, and *t*—time.

### 3.8. Molecular Dynamics

The following structure sets for all mutant forms and all dNTPs were used to perform molecular dynamics simulations: binary competent TdT-ssDNA with one Mg^2+^ ion—based on 4i2a crystal structure, ternary pre-catalytic TdT-ssDNA-dNTP with two active site Mg^2+^ ions—based on 4i27 crystal structure, and binary post-catalytic TdT-ssDNA + dN with two active site Mg^2+^ ions—based on 4i29 structure [18], all with the M6 ssDNA primer. Two additional elongated primer models were built only for the WT and F404W variant complexes: pre-catalytic TdT-ssDNA + 2dN-dNTP and post-catalytic TdT-ssDNA + 3dN. The models were based on crystal structures of murine TdT catalytic intermediates and human TdT AlphaFold structure [18,26], using Modeller 10.2 for Loop1 rebuilding [27]. Protonation states for histidine residues were calculated using the H++ server [28], with the H342 sidechain treated as protonated in the ternary complex models due to its positioning close to both the ssDNA phosphate backbone and dNTP triphosphate group. Simulation setup and simulations were performed in the GROMACS 2025 MD package [29]. The starting structures were placed in a dodecahedral periodic boundary cell, a classical computational approach to minimize edge effects in simulations. Explicit TIP3P model water and 50 mM of Cl^−^ and Na^+^ ions with Joung–Cheatham ion parameters were used to solvate and neutralize the system [30,31]. AMBER 14SB force field with OL24 DNA parameters was used to describe the protein and the DNA primer [32,33,34,35,36]. Parameters for the dNTP triphosphate group were obtained from [37], using R.E.D. Server 2.0 to calculate RESP charges for dNTPs [38]. An octahedral dummy model parameter set was employed for active site Mg^2+^ ions [39]. Cut-off for non-bonded interactions was set at 1.0 nm, with long-range electrostatic interactions treated using a smooth particle mesh Ewald method [40]. Covalent bonds involving hydrogen atoms were treated using LINCS solver, enabling a 2 ns timestep [41]. Steepest descent energy minimization was followed by NVT equilibration with V-rescale thermostat and NPT equilibration with C-rescale barostat conditions [42,43]. A leap-frog integrator with a 2 ns timestep was used for both equilibration and production simulations. Post-equilibration unrestrained MD simulations were run for 100 ns, obtaining 5 to 7 independent replicas for each system variant. Aggregate trajectories were processed using an integrated GROMACS 2025 toolset. Binding-free energy was estimated via the MMPBSA approach using the gmx_MMPBSA tool 1.6.4 [44,45].

## 4. Conclusions

In this study, we combined molecular dynamics simulations and experimental analyses to elucidate the roles of residues L397, F400, and F404 in the structure and function of human TdT. Our findings largely confirm and extend the functional picture established for the corresponding murine residues (L398, F401, and F405), underscoring the evolutionary conservation of their mechanistic roles. Our results demonstrate that L397 and F400 are critical for maintaining the integrity and rigidity of the dNTP-binding pocket, primarily through hydrophobic and stacking interactions that stabilize the catalytically competent conformation. The F404 residue, meanwhile, is essential for the proper architecture of the active site and for mediating effective interactions with both DNA and incoming nucleotides. Substitution of L397 or F400 with alanine increases the disorder and flexibility of Loop1, leading to impaired nucleotide incorporation, particularly for purine bases, but does not abolish activity entirely. The hydrogen bond between D472 and H474 residues, disrupted in the simulations of the F400A mutant, was previously noted as important for TdT function, with alanine substitution of any of these strictly conserved residues reducing template-independent activity [22]. These variants retain the ability to bind single-stranded DNA and can be partially rescued by Mn^2+^ ions, which are less strict in their coordination requirements.

The F404A variant completely abolishes terminal transferase activity. Both murine crystal structures and our simulations reveal that the F400A (F401A in mouse) variant exhibits a more disordered Loop1 compared to the F404A (F405A in mouse) variant and the WT protein. Notably, the crystal structure of the F405A DSB complex and simulations of the F404A binary competent complex show increasing disorder at the 3′ terminus of the primer, with the 3′ base unresolved in certain mispair contexts. In the F405A DSB complex, this disorder is partially mitigated by microhomology contacts with the template strand, but such stabilization is absent in the binary system [21]. This structural evidence supports the conclusion that the highly disordered Loop1 of the F400A variant still allows for ssDNA primer binding and elongation, whereas the F404A substitution prevents the formation of a catalytically competent complex altogether. Furthermore, the increased distance between the palm and fingers domains observed in F404A simulations correlates with the experimentally determined reduction in melting temperature, indicating global destabilization of the enzyme. Thus, the hierarchy of structural disruption—F404A/F405A causing the most severe global destabilization, and F400A/F401A primarily increasing Loop1 flexibility—appears to be conserved between human and murine TdT.

Experimentally, in the Mg^2+^ environment, the F404W variant exhibits reduced processivity in dTTP incorporation compared to the WT polymerase. Molecular dynamics simulations show that this substitution disrupts the thymine-specific π-π stacking interaction between the 3′ antepenultimate thymine base and the F404 side chain in the extended product complex, impairing the stabilization of polythymine-elongated ssDNA in the active site of this variant. While its increased observed nucleotide incorporation rate is matched by an increase in the population of catalytically favorable conformations during simulations of the extended pre-catalytic complex. This detailed functional and structural analysis of F404W in human TdT complements studies on aromatic substitutions at the equivalent position in murine TdT (F405Y/H), which also alter substrate specificity and processivity.

Additional support for this mechanistic model comes from recent findings on the murine F405H variant, which abolishes activity on ssDNA but retains reduced activity with ssRNA. In this case, the ribose backbone may form compensatory hydrogen bonds with the histidine side chain, a possibility not available to deoxyribose, further emphasizing the specificity of F404 for DNA substrates [23].

Taken together, our integrated structural, computational, and biochemical data highlight the critical importance of L397, F400, and F404 for catalytic efficiency and substrate specificity of TdT. The conservation of these functions across species confirms their fundamental role in the template-independent synthesis mechanism. These findings offer insights into the function of TdT and suggest a potential approach for engineering polymerase variants with uses in biotechnology.

## Figures and Tables

**Table 1:** Number of protein–primer hydrogen bonds and relative binding free energy estimation for the competent binary complex. Error values represent the standard error of the mean from independent trajectory replicates.

	WT	L397A	F400A	F404A	F404W
Number of H-bonds	8.0 ± 0.5	7.8 ± 0.6	7.5 ± 0.3	7.2 ± 0.2	7.9 ± 0.7
ΔGMMPBSA, kcal/mol	−85 ± 5	−76 ± 5	−77 ± 4	−64 ± 4	−85 ± 5

**Table 2:** Melting temperature of TdT WT and mutant forms.

	WT	L397A	F400A	F404A	F404W
T_m_, °C	46.2 ± 0.2	44.4 ± 0.4	46.2 ± 0.5	44.7 ± 0.4	46.0 ± 0.3

**Table 3:** Dissociation constants of nucleoside triphosphate (Flu-dUTP) and single-stranded DNA primer (FAM-M44).

Enzyme	*K*_d_, μM	
	Flu-dUTP	FAM-M44
WT	0.9 ± 0.1	0.88 ± 0.08
L397A	1.5 ± 0.2	0.97 ± 0.08
F400A	1.1 ± 0.1	0.98 ± 0.07
F404A	1.5 ± 0.2	1.10 ± 0.09
F404W	1.0 ± 0.1	1.0 ± 0.1

**Table 4:** Observed rate constants *k*_obs_ for the incorporation of the *n* + 1 nucleotide in the presence of Mn^2+^ ions.

*k*_obs_, s^−1^	dATP	dCTP	dGTP	dTTP
WT	0.5 ± 0.1	2.5 ± 0.5	4.3 ± 0.6	0.37 ± 0.05
L397A	0.06 ± 0.02	0.10 ± 0.01	0.10 ± 0.02	0.019 ± 0.006
F400A	0.011 ± 0.003	0.095 ± 0.009	0.23 ± 0.05	0.044 ± 0.006
F404W	0.5 ± 0.1	1.2 ± 0.1	7 ± 2	0.48 ± 0.04

**Table 5:** Observed rate constants *k*_obs_ for the incorporation of the *n* + 1 nucleotide in the presence of Mg^2+^ ions.

*k*_obs_, s^−1^	dATP	dCTP	dGTP	dTTP
WT	0.033 ± 0.003	0.014 ± 0.003	0.14 ± 0.02	0.009 ± 0.001
F404W	0.06 ± 0.01	0.018 ± 0.004	0.19 ± 0.02	0.033 ± 0.006

**Table 6:** Primers for site-directed mutagenesis.

Mutant Form	Sequence
L397A	Fwd 5′ GCCTAGCAGGAAGGTTGATGCTGCGGATCATTTTCAAAAGTGC 3′ Rev 5′ GCACTTTTGAAAATGATCCGCAGCATCAACCTTCCTGCTAGGC 3′
F400A	Fwd 5′ GCAGGAAGGTTGATGCTTTGGATCATGCTCAAAAGTGCTTTCTG 3′ Rev 5′ CAGAAAGCACTTTTGAGCATGATCCAAAGCATCAACCTTCCTGC 3′
F404A	Fwd 5′ GGATCATTTTCAAAAGTGCGCTCTGATTTTCAAATTGCCTCGTCAAAG 3′ Rev 5′ CTTTGACGAGGCAATTTGAAAATCAGAGCGCACTTTTGAAAATGATCC 3′
F404W	Fwd 5′ GGATCATTTTCAAAAGTGCTGGCTGATTTTCAAATTGCCTCGTCAAAG 3′ Rev 5′ CTTTGACGAGGCAATTTGAAAATCAGCCAGCACTTTTGAAAATGATCC 3′

**Table 7:** Oligonucleotides used as substrates in this study.

Mutant Form	Sequence
FAM-M44	5′ FAM-ATGCTATGGATTGATGTGACTAAGGTTGGAATGATGTGAAGAGA 3′
FAM-M6	5′ FAM-GGAAGA 3′

## Data Availability

The original contributions presented in this study are included in the article. Further inquiries can be directed to the corresponding author.
